# Variability and change in the hydro-climate and water resources of Iran over a recent 30-year period

**DOI:** 10.1038/s41598-020-64089-y

**Published:** 2020-05-04

**Authors:** Davood Moshir Panahi, Zahra Kalantari, Navid Ghajarnia, Samaneh Seifollahi-Aghmiuni, Georgia Destouni

**Affiliations:** 1 Department of Civil Engineering, Iran University of Science and Technology, Tehran, 13114-16846 Iran; 2 Department of Physical Geography and Bolin Centre for Climate Research, Stockholm University, SE-10691, Stockholm, Sweden

**Keywords:** Hydrology, Climate change

## Abstract

Comprehensive assessment of hydro-climatic variations and change trends is essential for understanding, mitigating, and adapting to key water resource changes in different parts of the world. We performed such an assessment on Iran, as representative of an arid/semi-arid and geopolitically important world region. We acquired and calculated data time series of surface temperature (T), precipitation (P), runoff (R), evapotranspiration (ET), and water storage change (DS), to determine their status and changes in and among the 30 main hydrological basins in Iran over the period 1986–2016. From 1986–2000 to 2001–2016, the country warmed, P mostly decreased and R even more so, while water storage was depleted (DS < 0) and ET increased in some basins. Overall, the extra water provided from primarily groundwater depletion has fed and kept ET at levels beyond those sustained by the annually renewable water input from P. This indicates unsustainable use of water for maintaining and expanding human activities, such as irrigated agriculture, in this part of the world.

## Introduction

Climate change and human activities can alter large-scale patterns of hydrological fluxes (precipitation, runoff, evapotranspiration)^1,2^ and deplete groundwater^3^ and surface waters^4,5^, with feedbacks to climate^6^. For example, the dramatic decline in Lake Urmia in Iran is a well-known destructive impact of hydrological change in this arid/semi-arid part of the world^5,7,8^. Other surface waters in Iran have also changed in recent decades, including Lake Maharloo-Bakhteghan in central Iran^9^, Lake Hamoon in the east^10^, and Lakes Shadegan and Hoor-Al-Azim in the south and southwest^11,12^. Moreover, hydrological changes in Iran are not limited to surface waters, but also extend to groundwater resources, with substantial decreases in groundwater level reported for different parts of the country^13^.

Surface and subsurface hydrological changes in Iran have previously been estimated for six major hydrological basins over a period of 11 years, based on general availability of remote sensing data for both surface water and groundwater^14^. Effects of surface water withdrawals on long-term average surface water storage have been estimated on finer spatial resolution (for 30 main hydrological basins in Iran), but without accounting for related groundwater changes^15^. Considering the extent and magnitude of water changes in Iran, and their impacts and consequences in such a dry and geopolitically important part of the world^13,16^, there remains a need for more long-term, comprehensive, but still relatively fine-resolution assessments of hydro-climatic variability and change in the country and the region. Such assessments are essential for understanding, mitigating, and adapting to key water resource changes. This study aims to address this need by determining annual conditions and changes in both surface and subsurface water resources in and across Iran’s 30 main hydrological basins over the recent 30-year period with relevant available data (1986–2016).

With this aim, we use empirical data from 103 synoptic meteorological stations on precipitation (P) and surface temperature (T)^17^, along with data from 213 hydrometric stations on runoff (R; measured discharge normalized to associated catchment area)^16^, and from reports on changes in water storage (DS) for each of the studied 30 main hydrological basins of Iran, including groundwater storage in the aquifers underlying each basin^13,18^ (see further Methods section). Actual evapotranspiration (ET), for which no direct basin-scale measurement data are available, is estimated from the measured P, R, and DS data and the fundamental basin-scale water balance equation:

1 $$ET=P-R-DS$$

Resulting ET values and their changes over the study period are analyzed in relation to the commonly considered Budyko space^19^, for further understanding and interpretation of basin-scale ET conditions and their temporal changes^2,20,21^. For assessment of changes in temporal and basin-average hydro-climatic conditions over the 30-year study period, we compare mean values of the water balance components and temperature in the first (1986–2000) and the second (2001–2016) half of the period 1986–2016. Furthermore, for assessment of variability and change trends over the whole period 1986–2016, a Mann-Kendall test^23,24^ is applied to country-average values of the hydro-climatic variables.

## Results and Discussion

### Long-term average conditions

**Figure 1:**
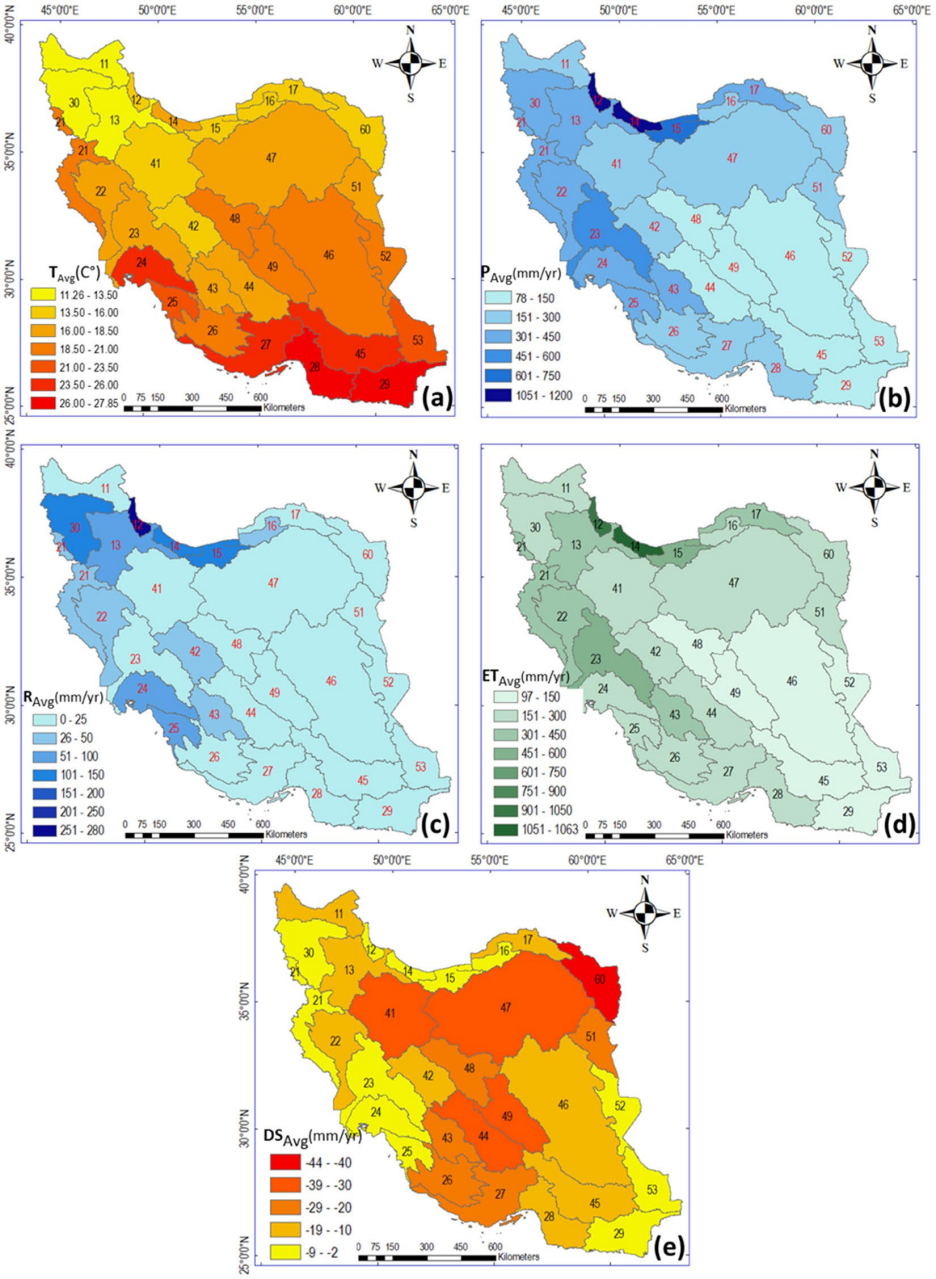
Long-term average values of water balance components and temperature in the 30 main basins in Iran, 1986–2016. (**a**) Temperature (T), (**b**) precipitation (P), (**c**) runoff (R), (**d**) evapotranspiration (ET), and (**e**) water storage changes (DS), The maps in this Figure are drawn by ArcGIS 10.6 (https://desktop.arcgis.com/en/arcmap).

Iran is located at longitude 25–40°N and latitude 44–65°E and over 94% of the country (total area 1.65 million km^2^ ^22^); has an arid/semi-arid climate (Fig. 1). The highest elevations are found in the Zagros and Alborz mountain ranges, which block moisture from the west and north, resulting in uneven distributions of T and P across the country (Fig. 1). The spatial-temporal average T over Iran and the whole study period 1986–2016 is 18.5 °C, with basin-average T being greater than 16 °C in 23 of the 30 study basins (Fig. 1a). The difference between the lowest (11.25 °C in north-west) and highest (27.85 °C in south) basin-average T is 16.65 °C (Fig. 1a). Mean annual P in the same period is 311 mm and the difference between the lowest and highest P is 1107 mm (Fig. 1b). In eight of the 30 basins, mean annual P is less than 150 mm and in 10 basins it is 150–300 mm, while it only exceeds 750 mm in two northern basins. The spatial differences of 16.65 °C for T and 1107 mm/year for P show the geographic diversity of hydro-climatic conditions in Iran, as also reported in previous studies^16^.

Mean annual runoff is on average among the basins about 39 mm, but is much lower, 0–25 mm, in 18 of the 30 basins (Fig. 1c). Basins with very low (<25 mm/year) or low (<39 mm/year) R have limited access to surface water resources, and rivers and lakes in those areas are particularly vulnerable to shifts in water balance.

Mean annual actual evapotranspiration, as calculated from the basin-scale water balance (Eq. 1), is 290 mm (Fig. 1d), which is close to mean annual P (311 mm). ET is highest, 1050–1063 mm/year, in basins 12 and 14, which also has the highest P (1050–1200 mm/year). The lowest ET values (150–197 mm/year) are found in central and southeastern basins of Iran, with mean P of 150–178 mm/year. Comparison of the ET and P maps across all basins (Fig. 1d and Fig. 1b) shows that the overall spatial pattern of ET follows that of P. In other words, the limiting factor for ET in Iran is the input of water through P, rather than the energy input related to T.

Based on statistics and reports from the Ministry of Energy of Iran^18^, total (surface and ground) water storage changes (DS) in Iran are on average negative over the study period (−16 mm/year) across all 30 basins, with 18 basins having mean annual decrease rates of more than −10 mm and 7 basins more than −30 mm (Fig. 1e). The negative mean DS in all basins implies a decline in water resource availability in Iran.

### Variability and change in temperature and water fluxes

For an overview of variability and change trends over the whole 30-year study period, Fig. S1 in Supplementary Material (SM) shows time series of country-average (area-weighted averaging of the basin-scale) values of the water balance components and temperature. Table 1 presents the results of the Mann-Kendall test^23,24^ applied to these time series, considering a 95% confidence level. Significant trends emerge for temperature T (increasing) and runoff R (decreasing), while the trends in P, ET and DS are non-significant.

**Table 1:** Results of Mann-Kendall test (95% confidence level) applied to trends in country-average water balance components and temperature.

	Temperature	Precipitation	Runoff	Evapotranspiration	Storage Change
P-Value	*0.011	0.053	*0.0002	0.563	0.277
Trend	Increase	Decrease	Decrease	Decrease	Decrease

*Significant trend at 95% confidence level.

**Figure 2:**
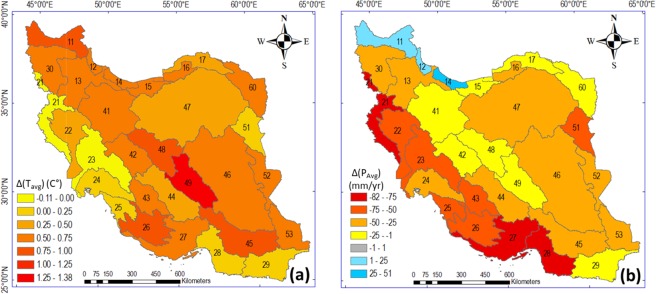
Change in (**a**) average temperature (T) and (**b**) average precipitation (P) in the 30 main basins in Iran between two 15-year sub-periods (1986–2000 and 2001–2016), The maps in this Figure are drawn by ArcGIS 10.6 (https://desktop.arcgis.com/en/arcmap).

**Figure 3:**
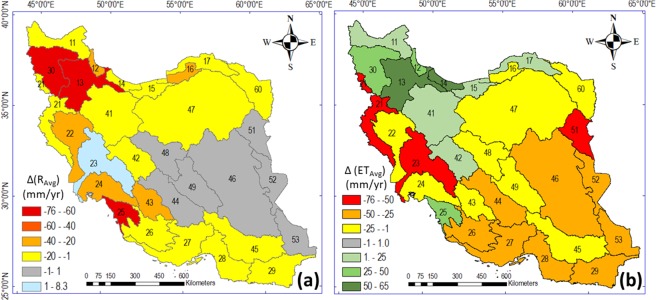
Change in (**a**) average runoff (R) and (**b**) average evapotranspiration (ET) in the 30 main basins in Iran between two 15-year sub-periods (1986–2000 and 2001–2016), The maps in this Figure are drawn by ArcGIS 10.6 (https://desktop.arcgis.com/en/arcmap).

Figure 2 further shows resulting shifts in period and basin average T and P, and Fig. 3 shows corresponding shifts in average ET and R from the first (1986–2000) to the second (2001–2016) 15-year sub-period within the total study period (see further Fig. S2 in SM for the average values of all variables in each 15-year sub-period). In addition to dividing the total study period into two equally long sub-periods for consistent comparison of conditions between them, the reasonableness of this sub-period division is also strengthened by a trend shift point emerging between these sub-periods, around year 2000, from trend analysis of the cumulative curve of runoff R (Fig. S3). Results also show that 28 of the 30 study basins have experienced an increase in period-average T, i.e., net warming, from 1986–2000 to 2001–2016 (Fig. 2a), in consistency with the significant T increase trend seen over the whole study period (Table 1). In 18 basins, the warming has been greater than 0.5 °C and in 8 basins it has been greater than 1 °C. The highest mean T in both sub-periods (27.73 °C in 1986–2000 and 27.97 °C in 2001–2016) is recorded in basin 28, in the south of the country.

Mean annual P has mostly decreased from 1986–2000 to 2001–2016, with decreases seen in all basins except 12, 13, and 14 (Fig. 2b), also in consistency with the non-significant decrease trend of country-average P seen over the whole study period (Table 1). The largest absolute decrease in P is 82 mm/year (66%), in basin 27 in the southwest. The greatest absolute increase in P is 51 mm/year (4%) in basin 14 in the north (see also Table S1 in SM). The western basins from north to south have experienced an average P decrease of around 65 mm/year, i.e., a 33 mm/year greater P decrease than the country average (32 mm/year). This is important, because these regions of Iran play an essential role in crop production^25^. The decrease of P in these regions may thus be a limiting factor for agricultural production, which can drive or exacerbate unsustainable exploitation of regional water resources.

With regard to runoff, the arid central and eastern basins of Iran have mean R of less than 25 mm/year (Fig. 1c), and this has not changed much from 1986–2000 to 2001–2016. Basin 23 is the only basin exhibiting an increase in R, which may be due to the construction and active management of multiple dams and surface reservoirs in this basin, regulating the release of water within and between years (see Fig. S4 in SM for the number and capacity of dams in this basin). All other basins have experienced a decrease in R, consistent with the significant decrease trend in country-average R seen over the whole study period (Table 1). At basin scale, basins 25, 13, and 30 exhibit the largest relative R decreases, of 243, 151, and 92%, respectively.

The fundamental basin-scale water balance (Eq. 1) implies that, in order to fully understand a change in R, given an associated change in the P water input, it is also necessary to consider the changes in ET and DS over the same period. The ET flux connects the land surface and the atmosphere, and changes in ET may be driven by both atmospheric climate change and human activities in the landscape^1,2,21^. No general pattern is observed for ET changes from 2001–2016 to 1986–2000 (Fig. 3b), even though there is a non-significant decrease trend in country-average ET in consistency with the non-significant decrease trend in country-average P (Table 1). In some northeastern, northwestern, and central basins (e.g., 42, 41, 13, 30, 17, 18, and 25), ET has increased in spite of decrease in P, which implies more water-limited conditions. In particular, the major decreases seen for R in basins 30, 13, and 25 are related to major ET increases, in addition to the P decreases occurring in the same basins. Major change is also seen in Fig. 3a for runoff in the Lake Urmia basin (number 30), a well-known example of dramatic hydrological change in Iran^5^. Specifically, Fig. 3a shows a massive decrease in R into Lake Urmia (dark red, decrease of 70 mm/year). Figure 3b further shows ET increase in this basin (dark green, increase of 42 mm/year). The R decrease, resulting from the increase in ET and decrease in P, is consistent with previous findings for the Lake Urmia basin^5,7,8^ and explains the dramatic shrinkage of Lake Urmia.

### Variability and change in water storage

**Figure 4:**
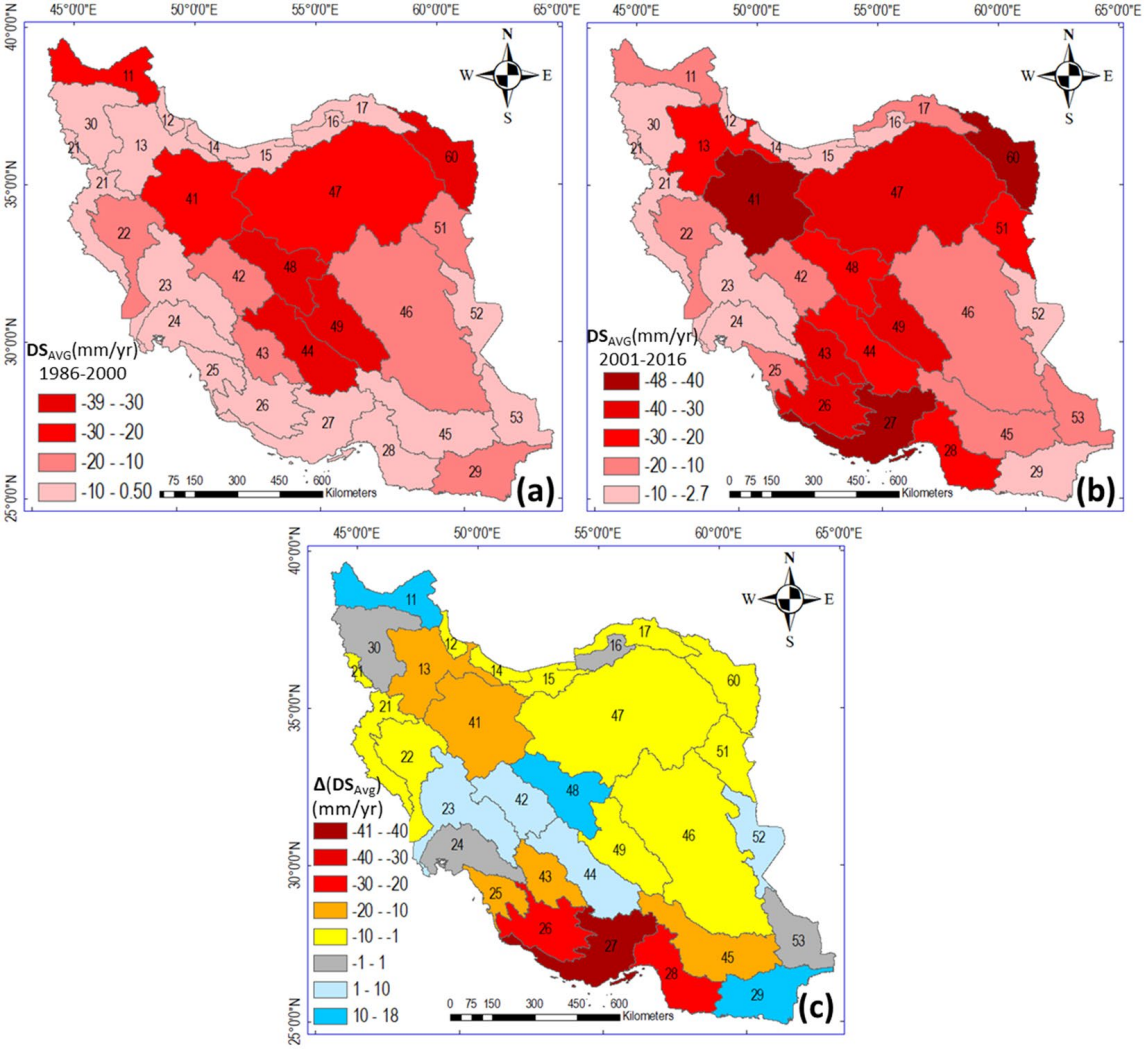
Change in mean annual water storage (DS) in the 30 study basins in Iran during (**a**) 1986–2000 and (**b**) 2001–2016, and (**c**) differences in the rate of DS between the two periods, the maps in this Figure are drawn by ArcGIS 10.6(https://desktop.arcgis.com/en/arcmap).

Water storage changes in groundwater and surface water typically occur seasonally within each year, with long-term average DS remaining around zero under sustainable use of water resources^1,2,20^. If temporal average DS in a hydrological basin systematically deviates from zero over many years, this implies systematic lowering (for DS < 0) or raising (for DS > 0) of average water levels over the whole basin. Such changes may be due to consumptive use of water by human activities removing water from the basin (e.g., by increased ET and atmospheric transfer of that water to other basins/regions), or by such activities adding water to the basin (e.g., through engineered water transfer from other basins/regions). In this study, the estimated DS values and their changes between the sub-periods in Iran include changes in both surface water and groundwater (Table 1, Fig. 4); see also the Methods section.

Overall, DS exhibits a non-significant decrease trend (Table 1) because it has been negative in Iran over the whole study period, with its decrease rate (i.e., the rate of water level decline) being more enhanced in the later sub-period (2001–2016; Fig. 4b) than in the earlier period (1986–2000; Fig. 4a) for 22 of the 30 basins. The decline rate enhancement between periods is greatest in basins 25, 26, 27, and 28 in the south (Fig. 4c). In eight basins (number 11, 23, 42, 44, 48, 16, 52, and 29), the DS decline rate is slower, but still negative, in 2001–2016. The systematic negative DS across Iran is likely due to human extraction of groundwater resources^15,26^. It primarily represents a decline in groundwater level, as this extends over the whole basin area, while surface waters cover only a small area fraction, although dramatic storage decreases have also occurred in surface water^5^. Regarding the particularly dramatic visible decline in Lake Urmia’s water level, note that Fig. 4a and b show average decline rate in the first and second sub-period, respectively, while Fig. 4c shows the shift in this rate between the periods. Since storage change in the Lake Urmia basin has been (and still is) negative and decreasing over the whole study period, the rate difference between sub-periods is relatively small.

**Figure 5:**
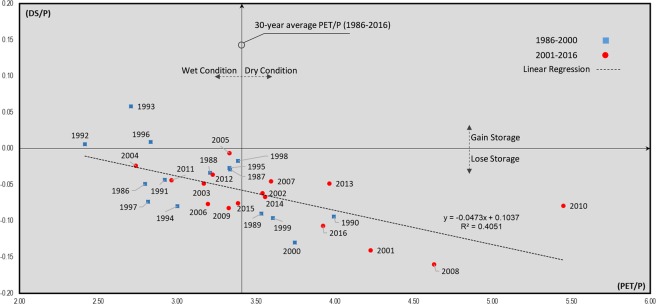
Water storage change to precipitation ratio (DS/P) plotted against the aridity index of potential evapotranspiration (PET) to precipitation ratio (PET/P). Data point values are for the whole of Iran, and the diagram is inspired by^27^.

Figure 5 further shows the ratio of annual DS rate to P (DS/P) versus the aridity index PET/P, where PET is the mainly T-dependent potential evapotranspiration (see Methods section) for the whole of Iran. The vertical black line in Fig. 5 represents the 30-year (1986–2016) country average PET/P, while data points to the right and left of this line represent years with drier and wetter conditions than the long-term country average, respectively. Data points above and below the horizontal axis represent years with positive and negative annual DS rate, respectively, with positive DS rate (storage increase) only seen in the years 1992, 1993, and 1996. The predominant decline in water storage across Iran is greater (DS more negative) for drier years (negative slope of regression line), but has also occurred under normal and wet conditions (with PET/P close to or lower than the long-term average value, respectively). The negative DS in all years of the second sub-period (red circles in Fig. 5) likely indicates increased human pressures and more unsustainable exploitation of water resources in Iran, as the country’s hydro-climatic conditions have mainly become drier in terms of both P and R from 1986–2000 to 2001–2016. However, unlike P, R, and DS, the ET conditions are not systematically drier across Iran in the later sub-period (Fig. 3b), for reasons explored further in the next section.

### Evapotranspiration shifts in Budyko space

**Figure 6:**
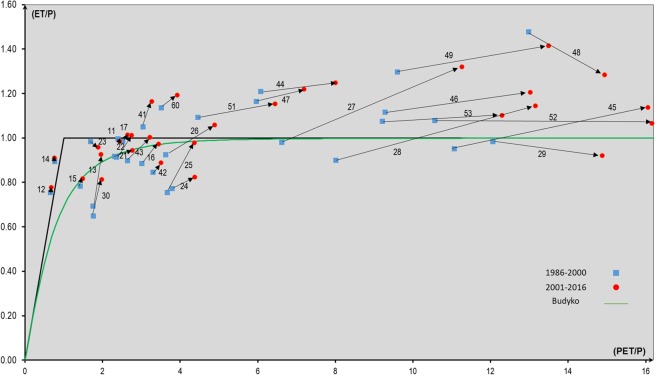
Budyko plot of conditions and changes in actual evapotranspiration (ET) versus potential evapotranspiration (PET) normalized to precipitation (P) in the 30 study basins in Iran in and between the sub-periods 1986–2000 and 2001–2016.

A Budyko space plot of the basin-wise conditions of ET/P versus PET/P, and shifts from 1986–2000 (blue symbols) to 2001–2016 (red symbols), provide some important insights regarding the fate of the extra water flux provided to the basins by the systematic water storage decline (negative DS) over Iran (Fig. 6). The green curve in the diagram represents the expected ET/P for the prevailing PET/P value in each hydrological basin, based on the commonly used Budyko equation(19), see also Table S2 in SM. Furthermore, the thick black line in the range PET/ P < 1 shows ET = PET as a theoretical upper limit of ET; data points of ET ≤ PET for PET/P < 1 indicate basins with energy-limited ET (Fig. 6). For PET/P > 1, the horizontal thick black line shows instead ET = P as a theoretical upper limit of ET; data points of ET ≤ P for PET/ P ≥ 1 should thus indicate basins with water-limited ET. However, the limit of long-term average ET = P assumes and applies for long-term average DS = 0. At negative DS, extra water is released from storage as this declines and feeds into the main water fluxes ET and/or R, in addition to the input water provided from P. This water addition is why ET/P can exceed the assumed long-term average limit ET = P (Fig. 6) if DS < 0, as it is across Iran (Fig. 4).

With P, R, and DS obtained directly from reported measured data for the main Iranian basins and their underlying aquifers, ET is the only unknown variable (not directly given from reported measurements) in the water balance equation for each basin. As such, ET is fully constrained and can be directly calculated from the measured P, R, and DS data in that equation. In particular, the finding of increased ET (green basins, Fig. 3b) where both P and R have decreased (yellow to red basins, Figs. 2b and 3a, respectively) implies that the extra water provided from the systematically negative DS (Fig. 4) feeds predominantly into and increases ET in these basins, particularly in the recent period 2001–2016 (red symbols, Fig. 6) compared with the earlier period 1986–2000 (blue symbols, Fig. 6). Previous studies have also found considerable ET increases by human land- and water-use developments, such as irrigation and construction of dams and water reservoirs^1^, as well as agricultural expansion and intensification even without irrigation^2^. The results of our study show that the ET increase is even greater if water extracted from storage (DS < 0) is used for these developments, in addition to the annually renewable P input water.

## Conclusions

Over the 30-year study period, the main hydrological basins in Iran have warmed and their precipitation, runoff, and water storage have mostly decreased, while evapotranspiration has decreased in some basins and increased in others. In all basins, the extra water (primarily groundwater) provided from the water storage depletion has gone to maintain evapotranspiration beyond the levels sustained by the annually renewable water input from precipitation. Some basins with decreased precipitation and runoff still exhibit increased evapotranspiration, fed by extra water provided from even faster storage depletion.

Overall, these results indicate unsustainable use of water for maintaining and expanding human activities in this arid/semi-arid part of the world. If left uncontrolled, such excess use of water can have serious environmental and socio-economic consequences. This comprehensive assessment of Iran’s hydrological conditions and their changes over time advances understanding of water changes and their drivers, impacts, and feedbacks, in Iran and more generally. Based on the methodology used for this assessment and the results obtained, management strategies can be formulated for mitigating adverse water changes and their impacts, and improving water conditions, in Iran and similar arid/semi-arid regions of the world.

## Materials and Methods

Iran has 30 main hydrological basins range in area from 226 533 km^2^ (basin 47) to 7036 km^2^ (basin 12) with an average value of 90,122 km^2^. See Figure S5 and Table S1 in SM for more basin details. In order to investigate the hydro-climatic conditions and changes in these 30 basins over the 30-year study period 1986–2016 with relevant available data, we use the fundamental basin-scale water balance equation (Eq. 1). In addition to the availability of measured data with good temporal and spatial coverage, a main reason for selecting 1986–2016 as the study period is that major human activity development has also occurred in this period^16,28^ and we aim to investigate hydrological effects of such developments. The criterion for selecting the 103 synoptic meteorological and 213 hydrometric stations is access to at least 31 years of monthly data. In addition to the data series length criterion, selection of synoptic and hydrometric stations is also based on obtaining good representativeness and reliable data across all basins. The spatial distribution of the synoptic and hydrometric stations used is shown in Figure S6 in SM.

Thiessen polygons generated on the selected synoptic stations are used to determine mean monthly and annual T and P for each basin. To calculate R, each main basin is further subdivided into independent sub-basins based on the river network system and hydrometer stations at the outlets of these sub-basins. By dividing the measured discharge values by associated sub-basin area, R is calculated for each sub-basin and, using area-weighted averaging, the total basin-average R time series is calculated for each main basin. In total, 238 sub-basins are considered in the calculations of R for the 30 main basins of Iran. The division into sub-basins is shown in Figure S6 in SM.

Annual water storage change, DS, includes groundwater (GW) and surface water (SW) storage, but not soil moisture changes. Soil moisture is not considered in the assessment of subsurface storage changes because the soil moisture changes are much smaller than those in groundwater (see SM section: Soil moisture calculations), as also found in other studies^29,30^. Additionally, the correlation between ET and soil moisture variations has also been found to be relatively small, even in extreme events^31–33^.

Regarding GW and SW changes, hydrographs provided by^13^ and statistical reports from the Ministry of Energy (responsible for Water Sector Organizations)^18^ are used to calculate them, respectively. The GW changes are based on area-weighted averaging of groundwater level and volume changes in the aquifers underlying each basin. The boundaries of aquifers do not follow basin boundaries, but groundwater storage changes have been independently assessed and reported at basin scale^18^. Fig. S7 shows a map of Iran’s aquifers and how they underlie and are shared between basins. The aquifer area shared between any two main basins is just 5.3% of the total aquifer area below the land surfaces of the 30 main basins, implying that exchange fluxes of groundwater between boundaries are minor compared to the main water flux and groundwater storage changes occurring over each of the 30 large main basins studied here.

SW storage changes are further calculated based on observed data on annual surface water level and volume changes. In areas with no data on surface water storage, the contribution of surface water to total storage volume changes is considered to be close to zero (given the much smaller area extent of surface water in each basin relative to the groundwater underlying the whole basin). Estimated changes in GW storage are added to changes in SW storage, to give the overall volumetric changes in each basin. After calculating the annual P, R, and DS time series, ET is the only remaining unknown component in the water balance equation, which is then calculated using (Eq. 1) for each main basin.

To assess and illustrate the prevailing hydro-climatic conditions in each basin and their geographic distribution among basins over the whole study period 1986–2016, long-term average values are calculated for T and the components of the water balance equation (Fig. S1). Furthermore, the Mann-Kendall trend test^23,24^ is applied at 95% confidence level to the time series of country-average (area-weighted basin average) values of these hydro-climatic variables to assess their change trends over the whole study period.

To determine shifts in period-average conditions, the whole study period is further divided into the two equally long comparative sub-periods, 1986–2000 and 2000–2016, with a clear trend shift point for runoff also emerging between these sub-periods from the cumulative curve of R over Iran (Fig. S3). Basin-average T and water balance components are calculated for each sub-period to determine the possible shifts in their mean values between the sub-periods.

Finally, the Budyko space plot is created to assess shifts in basin-average ET/P and PET/P between the sub-periods. The basin-average values of these variables in the first and second sub-period are calculated plotted as corresponding points connected by arrows for each basin, to illustrate basin-specific shift magnitude and direction.

## Supplementary information

Supplementary Information.
