# Supplementary material for: Variability and change in the hydro-climate and water resources of Iran over a recent 30-year period
Source: Sci Rep. 2020 May 4;10:7450. doi: 10.1038/s41598-020-64089-y (PMC7198531; doi:10.1038/s41598-020-64089-y)
Supplement: Supplementary file 1 — Supplementary Information. [file 41598_2020_64089_MOESM1_ESM.docx]

**Supplementary Material**

**Variability and change in the hydro-climate and water resources of Iran over a recent 30-year period**

Davood MoshirPanahi^1,2^*, Zahra Kalantari^2^, Navid Ghajarnia^2^, Samaneh Seifollahi-Aghmiuni^2^, Georgia Destouni^2^

^1^Department of Civil Engineering, Science and Technology University of Iran, Tehran, Iran.

*Email: Davood.moshir@gmail.com

^2^Department of Physical Geography and Bolin Centre for Climate Research, Stockholm University, SE-10691 Stockholm, Sweden.

Contents (Reference numbering in all parts follows that in the main reference list):

- Table S1: Basin area, absolute temperature and precipitation changes, and relative precipitation change in the 30 main basins of Iran between the two sub-periods studied (1986-2000 and 2001-2016)
- Table S2. Budyko space vector length, ranking, and average for the 30 main basins in Iran in the two sub-periods studied (1986-2000 and 2001-2016)
- Figure S1. Time series of water balance equation components and temperature over Iran, 1986-2016. a) Temperature, b) Precipitation, c) Runoff, d) Evapotranspiration, and e) Storage Change.
- Figure S2. Average of all parameters in the 30 main basins in Iran in the two sub-periods studied (1986-2000 and 2001-2016).
- Figure S2 (continued). Average of all parameters in the 30 main basins in Iran in the two sub-periods studied (1986-2000 and 2001-2016).
- Figure S3. Accumulative runoff curve for Iran, 1986-2016.
- Figure S4. Number and capacity of dams built in 23 major basins in Iran during the study period (1986-2016).
- Figure S5. Relief map of Iran showing the location of the 30 main basins.
- Figure S6. Map of Iran showing the spatial distribution of synoptic and hydrometric stations providing data for the present analysis and the subdivision into sub-basins used for calculating surface runoff.
- Supplementary Material (SM) section: Soil moisture calculations
- Table .S3. Examples of soil parameters in Eq. S1 for two contrasting soil types (sand and clay loam) (Destouni and Verrot, 2014), and associated Δθ and Δ(DSsm) results calculated from Eq. S2 and Eq. S3, respectively, for three examples of runoff values R1 and R2 in Eq. S2

**Table S1: Basin area, absolute temperature and precipitation changes, and relative precipitation change in the 30 main basins of Iran between the two sub-periods studied (1986-2000 and 2001-2016)**

| **Basin Code** | **Area**  **(km^2^)** | **Average** | | **Absolute change** | |
| --- | --- | --- | --- | --- | --- |
|  |  | **Temperature**  **(C°)** | **Precipitation**  **(mm/year)** | **Temperature**  **(C°)** | **Precipitation**  **(mm/year)** |
| 11 | 39778 | 11.26 | 279.61 | 0.84 | 9.84 |
| 12 | 7036 | 13.82 | 1186.64 | 0.68 | 7.74 |
| 13 | 59194 | 12.19 | 384.70 | 0.51 | -30.95 |
| 14 | 10893 | 16.16 | 1178.33 | 0.7 | 50.94 |
| 15 | 18774 | 15.80 | 605.68 | 0.73 | -3.29 |
| 16 | 12986 | 15.38 | 266.93 | 0.63 | -26.63 |
| 17 | 26395 | 13.85 | 304.86 | 0.36 | -7.65 |
| 21 | 39297 | 18.84 | 414.52 | -0.08 | -78 |
| 22 | 51912 | 17.87 | 388.26 | 0.32 | -51.65 |
| 23 | 66676 | 17.76 | 549.27 | -0.11 | -64.3 |
| 24 | 40820 | 24.57 | 339.90 | 0.02 | -48.66 |
| 25 | 21309 | 23.08 | 322.51 | 0.19 | -53.36 |
| 26 | 47802 | 20.71 | 273.18 | 0.76 | -70.99 |
| 27 | 62896 | 24.61 | 164.34 | 0.56 | -81.75 |
| 28 | 44793 | 27.85 | 160.00 | 0.25 | -76.72 |
| 29 | 48524 | 26.24 | 111.84 | 0.09 | -23 |
| 30 | 51762 | 11.95 | 376.65 | 0.63 | -28.05 |
| 41 | 92884 | 15.08 | 267.75 | 0.6 | -10.25 |
| 42 | 41552 | 15.76 | 258.52 | 0.64 | -6.5 |
| 43 | 31452 | 17.65 | 334.87 | 0.53 | -56.14 |
| 44 | 57125 | 17.05 | 135.95 | 0.48 | -33.76 |
| 45 | 693475 | 25.47 | 109.20 | 0.76 | -36.61 |
| 46 | 206354 | 20.35 | 102.91 | 0.75 | -30.85 |
| 47 | 226533 | 18.13 | 152.95 | 0.46 | -25 |
| 48 | 48599 | 19.61 | 77.65 | 0.92 | -7.22 |
| 49 | 507366 | 18.88 | 91.83 | 1.38 | -24.52 |
| 51 | 33086 | 16.34 | 171.33 | 0.01 | -62.78 |
| 52 | 33589.6 | 20.99 | 90.05 | 0.61 | -35.29 |
| 53 | 36507 | 22.04 | 115.52 | 0.74 | -29.11 |
| 60 | 44295 | 15.61 | 234.79 | 0.61 | -18.02 |

**Table S2. Budyko space vector length, ranking, and average for the 30 main basins in Iran in**

**the two sub-periods studied (1986-2000 and 2001-2016)**

| **Basin code** | **1986-2000** | | **2001-2016** | | **Vector length** | **Rank** |
| --- | --- | --- | --- | --- | --- | --- |
|  | **PET/P** | **ET/P** | **PET/P** | **ET/P** |  |  |
| 11 | 2.4 | 1 | 2.43 | 0.99 | 0.04 | 28 |
| 12 | 0.65 | 0.75 | 0.67 | 0.78 | 0.03 | 29 |
| 13 | 1.76 | 0.69 | 1.96 | 0.93 | 0.31 | 21 |
| 14 | 0.76 | 0.89 | 0.76 | 0.91 | 0.02 | 30 |
| 15 | 1.42 | 0.78 | 1.49 | 0.82 | 0.08 | 27 |
| 16 | 3.01 | 0.89 | 3.45 | 0.97 | 0.45 | 18 |
| 17 | 2.53 | 0.98 | 2.65 | 1.01 | 0.12 | 26 |
| 21 | 2.3 | 0.92 | 2.77 | 0.94 | 0.47 | 17 |
| 22 | 2.36 | 0.91 | 2.74 | 1.01 | 0.39 | 20 |
| 23 | 1.69 | 0.98 | 1.89 | 0.96 | 0.2 | 25 |
| 24 | 3.79 | 0.77 | 4.38 | 0.82 | 0.59 | 15 |
| 25 | 3.66 | 0.75 | 4.36 | 0.98 | 0.73 | 14 |
| 26 | 3.63 | 0.92 | 4.89 | 1.06 | 1.27 | 12 |
| 27 | 6.62 | 0.98 | 11.26 | 1.32 | 4.65 | 4 |
| 28 | 8.01 | 0.9 | 13.16 | 1.14 | 5.15 | 2 |
| 29 | 12.06 | 0.98 | 14.88 | 0.92 | 2.82 | 8 |
| 30 | 1.77 | 0.65 | 1.98 | 0.81 | 0.27 | 22 |
| 41 | 3.05 | 1.05 | 3.27 | 1.16 | 0.26 | 23 |
| 42 | 3.3 | 0.85 | 3.5 | 0.89 | 0.21 | 24 |
| 43 | 2.64 | 0.9 | 3.22 | 1 | 0.58 | 16 |
| 44 | 6.08 | 1.21 | 8 | 1.25 | 1.92 | 11 |
| 45 | 11.07 | 0.95 | 16.05 | 1.14 | 4.99 | 3 |
| 46 | 9.28 | 1.12 | 13.02 | 1.21 | 3.74 | 6 |
| 47 | 5.96 | 1.16 | 7.19 | 1.22 | 1.23 | 13 |
| 48 | 12.98 | 1.48 | 14.94 | 1.28 | 1.97 | 10 |
| 49 | 9.6 | 1.3 | 13.5 | 1.42 | 3.89 | 5 |
| 51 | 4.46 | 1.09 | 6.45 | 1.15 | 1.99 | 9 |
| 52 | 10.56 | 1.08 | 16.16 | 1.07 | 5.6 | 1 |
| 53 | 9.21 | 1.07 | 12.29 | 1.1 | 3.08 | 7 |
| 60 | 3.51 | 1.14 | 3.92 | 1.19 | 0.42 | 19 |

**Figure S1. Time series of water balance equation components and temperature over Iran, 1986-2016. a) Temperature, b) Precipitation, c) Runoff, d) Evapotranspiration, and e) Storage Change.**

**
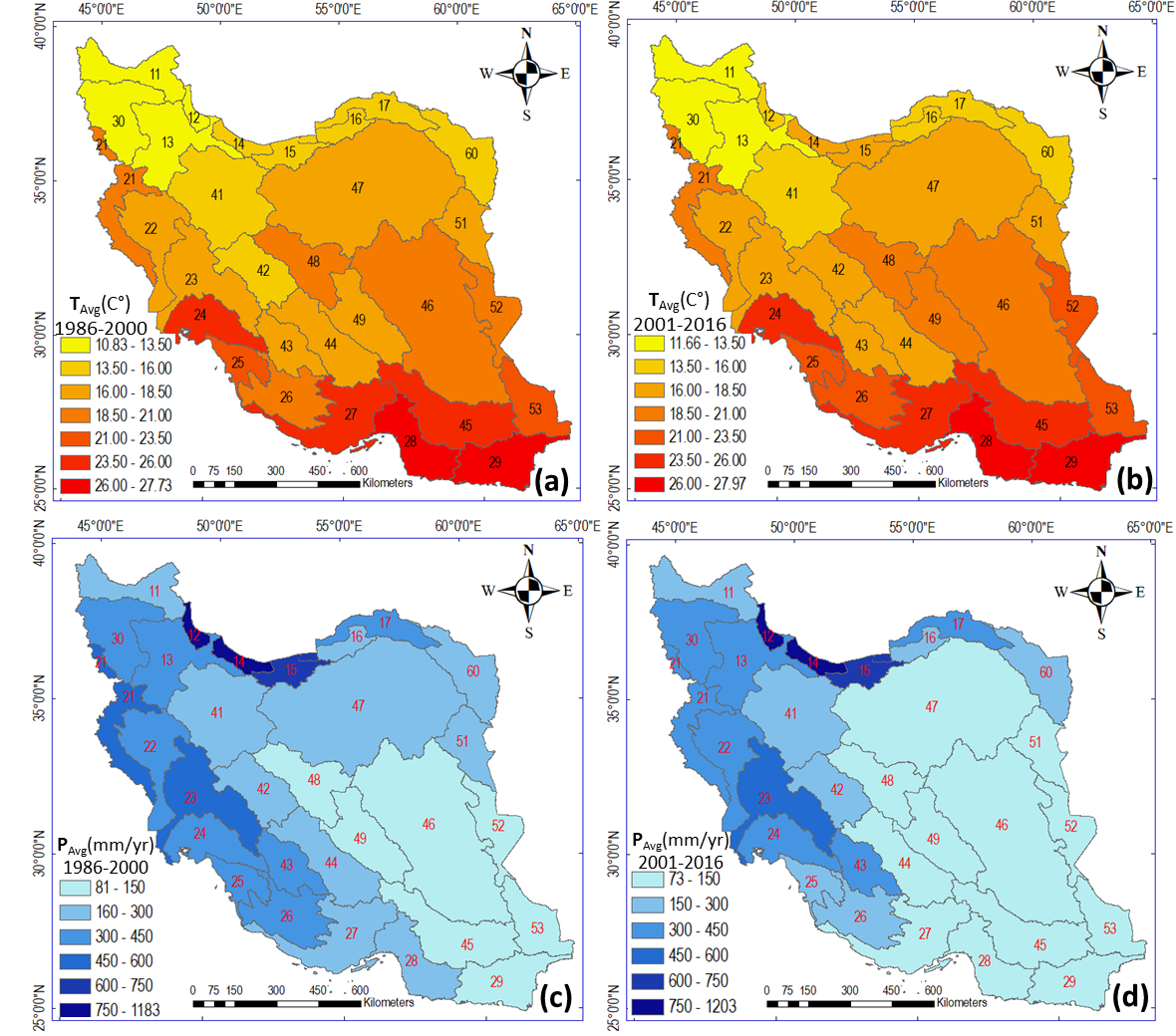
**

**Figure S2. Average of all parameters in the 30 main basins in Iran in the two sub-periods studied (1986-2000 and 2001-2016), The maps in this Figure are drawn by ArcGIS 10. 6(https://desktop.arcgis.com/en/arcmap).**

**
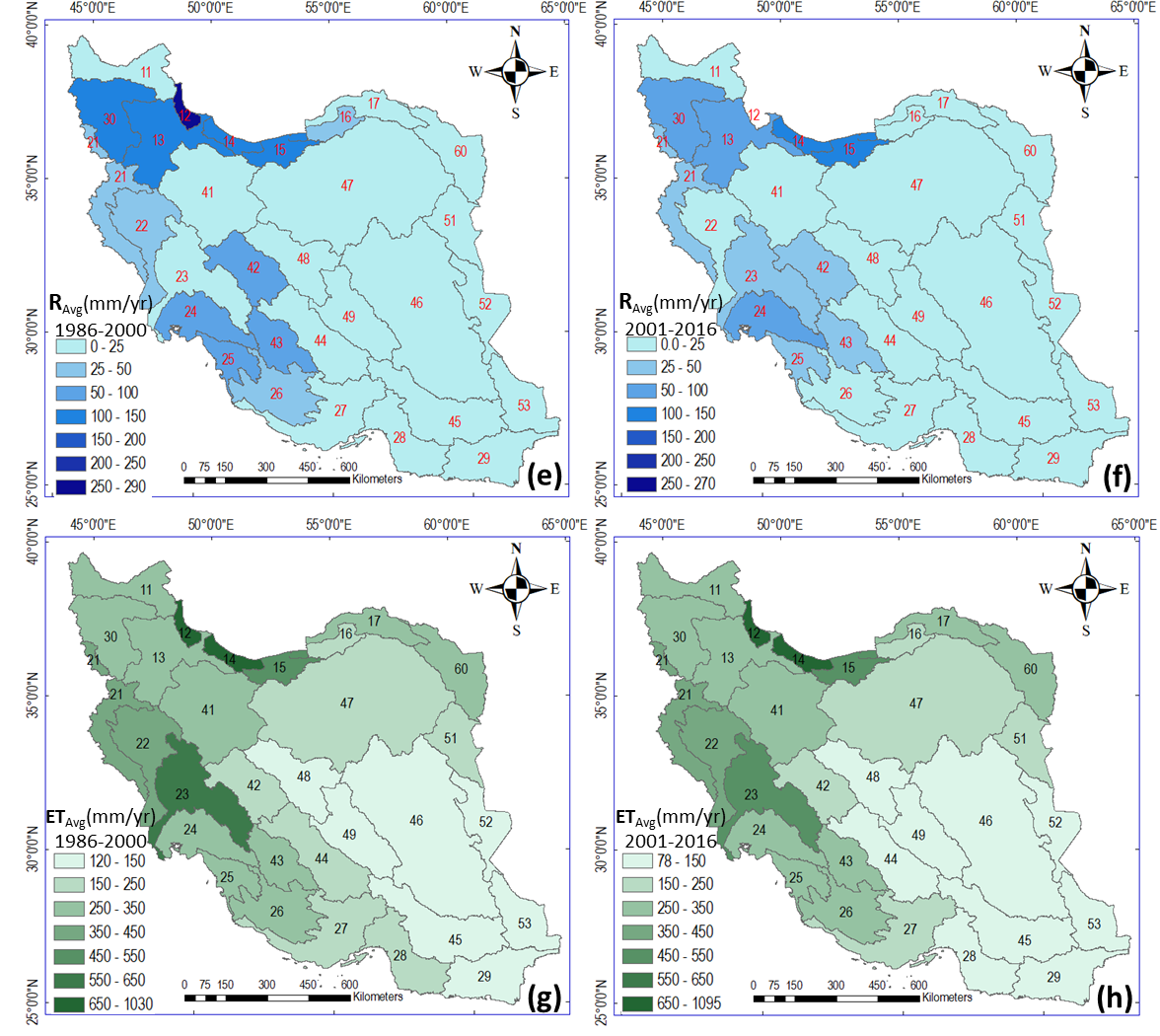
**

**Figure S2 (continued). Average of all parameters in the 30 main basins in Iran in the two sub-periods studied (1986-2000 and 2001-2016), The maps in this Figure are drawn by ArcGIS 10.6(https://desktop.arcgis.com/en/arcmap).**


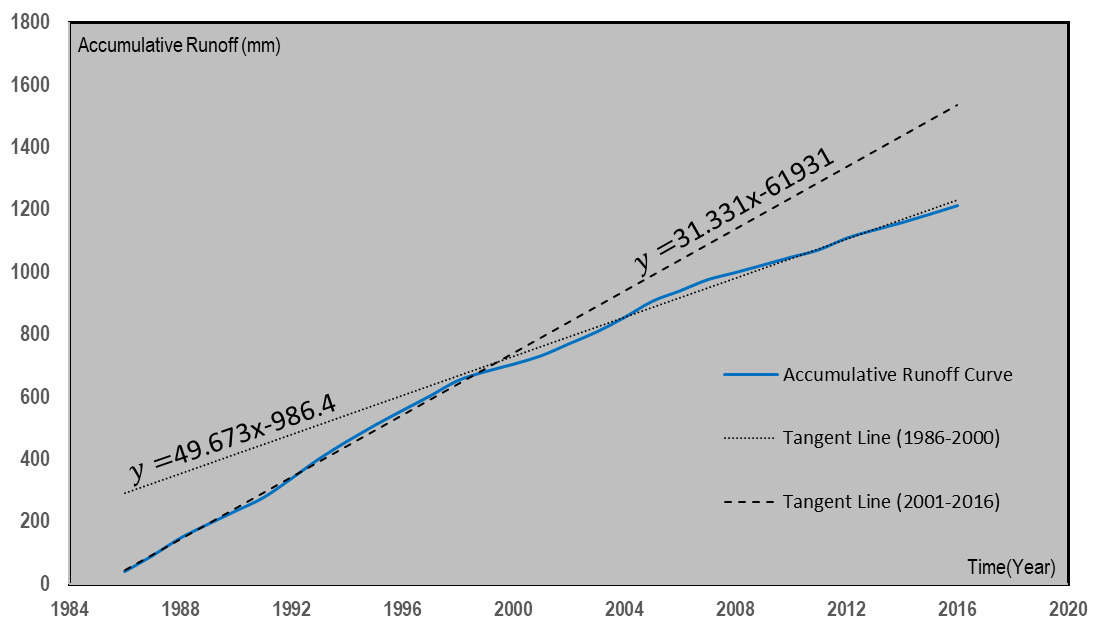


**Figure S3. Accumulative runoff curve for Iran, 1986-2016.**

**
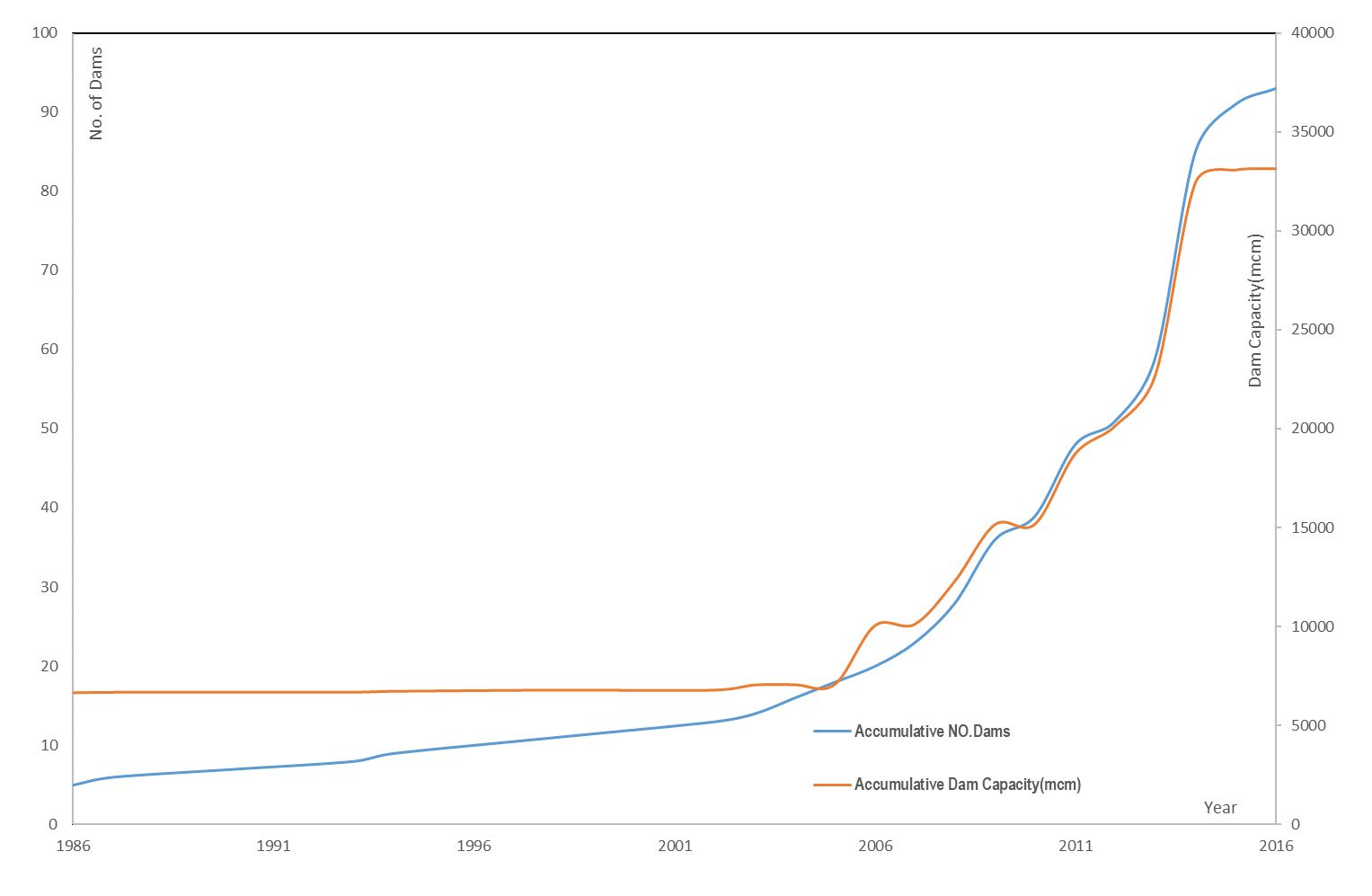
**

**Figure S4. Number and capacity of dams built in 23 major basins in Iran during the study period (1986-2016).**

**
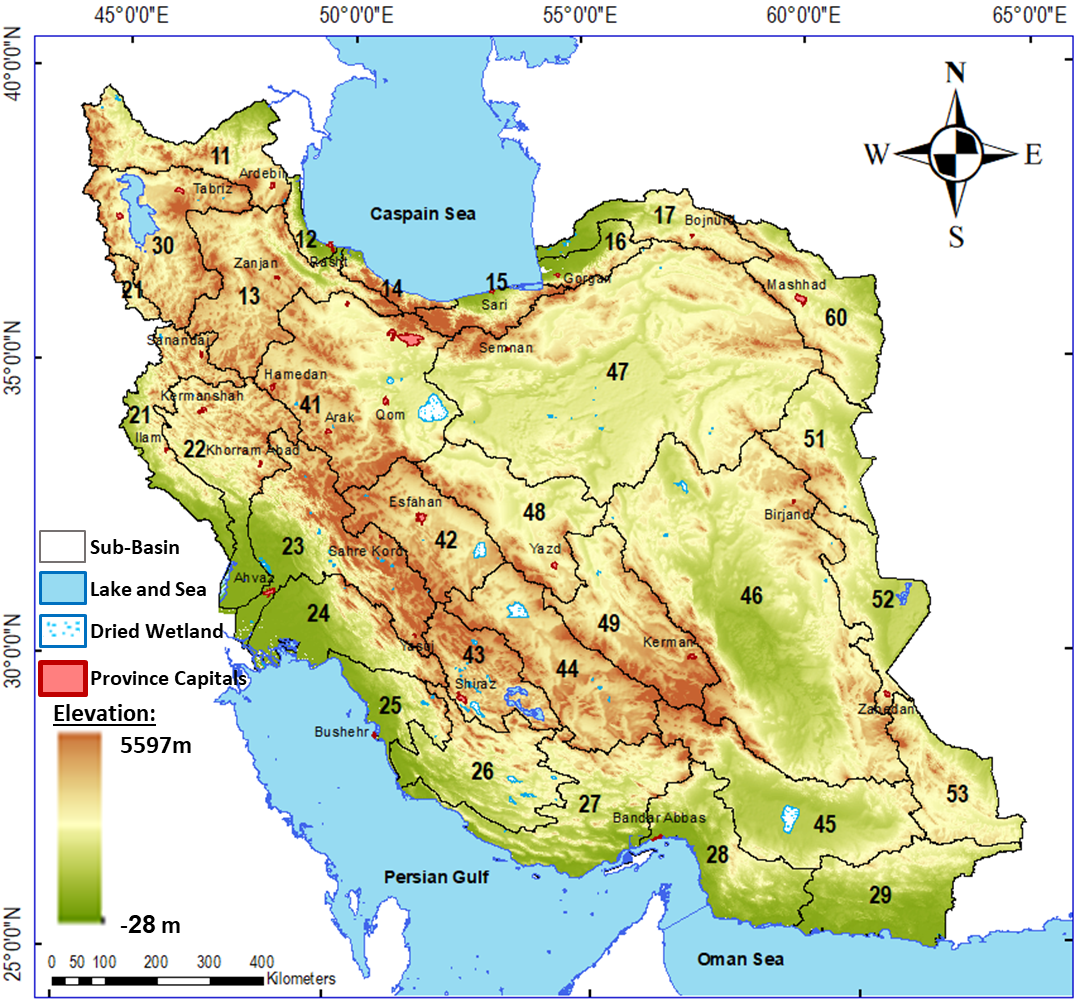
**

**Figure S5. Relief map of Iran showing the location of the 30 main basins. The maps in this Figure are drawn by ArcGIS 10.6(https://desktop.arcgis.com/en/arcmap).**

**
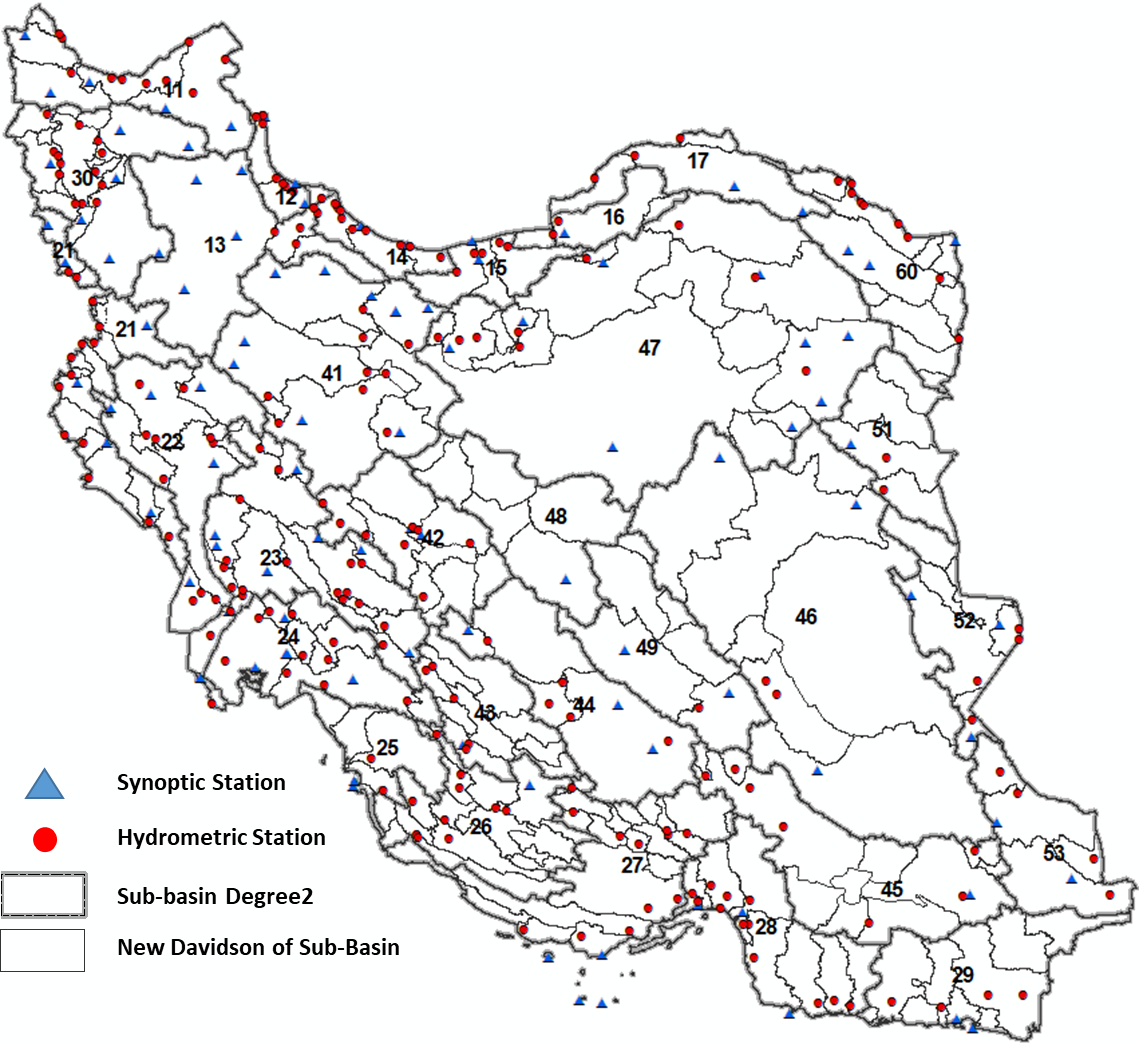
**

**Figure S6. Map of Iran showing the spatial distribution of synoptic and hydrometric stations providing data for the present analysis and the subdivision into sub-basins used for calculating surface runoff. The maps in this Figure are drawn by ArcGIS 10.6(https://desktop.arcgis.com/en/arcmap).**


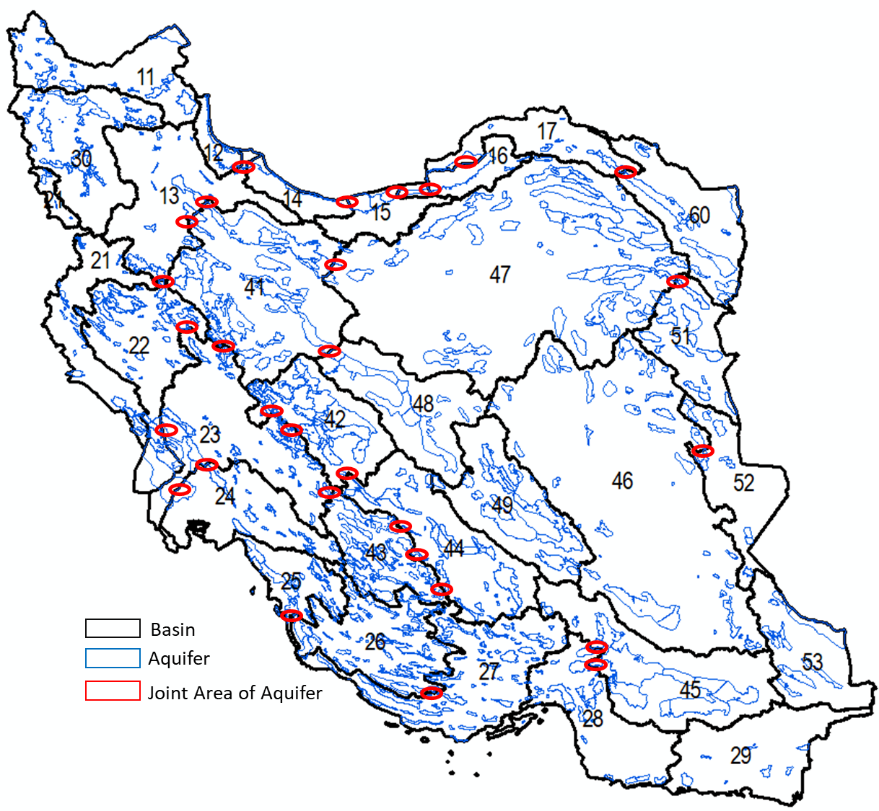


**Figure S7. Map of Iran’s aquifers, basins, and aquifers shared between basins. The maps in this Figure are drawn by ArcGIS 10.6(https://desktop.arcgis.com/en/arcmap).**

**Supplementary Material (SM) section: Soil moisture calculations**

We assess here how much soil moisture change (DS_sm_) can likely contribute to total DS in the basin- and period-average annual water balance (Eq. 1), for comparison with the corresponding contribution of groundwater storage change to DS (main Fig. 4, SM Fig. S1e). To do this, we can, e.g., use the expression of area- and depth-average volumetric soil water content θ from Destouni and Verrot (2014):

$\theta=\left( \frac{\alpha R}{K_{s}} \right)^{\beta}\left( \theta_{s}-\theta_{ir} \right)-\theta_{ir}=\left( \frac{\theta_{s}-\theta_{ir}}{K_{s}^{\beta}} \right){(\alpha R)}^{\beta}-\theta_{ir}\equiv{A\cdot(\alpha R)}^{\beta}-\theta_{ir}$ (Eq. S1)

where α is the long-term average fraction of total runoff R following the pathway of infiltration and percolation through the soil to groundwater recharge and flow into surface water runoff. Furthermore, K_s_ is saturated hydraulic conductivity, θ_s_ is water content at saturation, θ_ir_ is residual (irreducible) water content, and β is a soil characteristic parameter linked to the pore size distribution of different soil types; as θ, all soil parameters represent area- and depth-average values (Destouni and Verrot, 2014).

From Eq. S1, it follows that a shift inR, such as that from 1986-2000 (period 1 with R=R_1_) to 2001-2016 (period 2 with R=R_2_) in Iran, leads to the following shift in θ (with the fraction α considered, for simplicity, to remain more or less the same between the periods):

$\Delta\theta=\theta_{2}-\theta_{1}=A\alpha^{\beta}\left( R_{2}^{\beta}-R_{1}^{\beta} \right)$ (Eq. S2)

Furthermore, applying Δθ over a soil depth of z=3.5 m (3.5m is assumed based on root zone depth from different reanalysis and climate model) yields the shift in period-average annual rate of storage change related to soil moisture, Δ(DS_sm_), from 1981-2000 to 2001-2016 as:

$\Delta{(DS}_{sm})=\frac{\Delta\theta\cdot*3.5m}{15 years}$ (Eq. S3)

Table S3 lists some typical values of the soil parameters in Eq. S1 for two contrasting soil type examples (sand and clay loam), used also by Destouni and Verrot (2014), and associated Δθ and Δ(DS_sm_) results from Eq. S2 and Eq. S3, respectively, for examples of relevant R_1_ and R_2_ values for the mostly drying runoff conditions between the two sub-periods in Iran. In these calculations, the fraction α is set to its maximum value α=1, in order not to underestimate the influence of the shift in R on Δθ and Δ(DS_sm_).

Across all calculation examples, the resulting shift Δ(DS_sm_) in average annual rate of storage depletion due to decrease in period-average soil moisture from 1981-2000 to 2001-2016 ranges from -1.2 to -1.8 mm/year (Table S3). In comparison, the corresponding shift in total water storage ∆(DS), estimated only based on groundwater storage change, is overall much greater (ranging to around -40 mm/year, Figure 4c). This comparison justifies the study focus on the groundwater contribution to total DS and its inter-period shift ∆(DS).

**Table S3. Examples of soil parameters in Eq. S1 for two contrasting soil types (sand and clay loam) (Destouni and Verrot, 2014), and associated Δθ and Δ(DSsm) results calculated from Eq. S2 and Eq. S3, respectively, for three examples of runoff values R1 and R2 in Eq. S2.**

|  | **Sand** | | **Clay loam** | |
| --- | --- | --- | --- | --- |
| **K_s_** (m/s) | 9.3 ⋅ 10^-5^ | | 1.2 ⋅ 10^-5^ | |
| **K_s_** (m/year) | 2933 | | 378 | |
| **θ_s_** | 0.45 | | 0.40 | |
| **θ_ir_** | 0.02 | | 0.15 | |
| **β** | 0.18 | | 0.11 | |
| **A** (for K_s_ in m/year) | 0.1022 | | 0.1301 | |
|  | **R_1_** (m/year) | **R_2_** (m/year) | **R_1_** (m/year) | **R_2_** (m/year) |
| **Example 1** | 0.05 | 0.03 | 0.05 | 0.03 |
| **Δθ** | -0.0052 | | -0.0051 | |
| **Δ(DS_sm_)** (m/year) | -0.0012 | | -0.0012 | |
| **Example 2** | 0.10 | 0.05 | 0.10 | 0.05 |
| **Δθ** | -0.0079 | | -0.0074 | |
| **Δ(DS_sm_)** (m/year) | -0.0018 | | -0.0017 | |
| **Example 3** | 0.20 | 0.13 | 0.20 | 0.13 |
| **Δθ** | -0.0057 | | -0.0050 | |
| **Δ(DS_sm_)** (m/year) | -0.0013 | | -0.0012 | |
|  | | | | |

**Section References**

- Destouni, G., and Verrot, L. (2014). Screening long-term variability and change of soil moisture in a changing climate. J. Hydrol., 516, 131-139. <https://doi.org/10.1016/j.jhydrol.2014.01.059>
